# Thermal and Thermo-Mechanical Properties of Poly(L-lactic Acid) Biocomposites Containing β-Cyclodextrin/d-Limonene Inclusion Complex

**DOI:** 10.3390/ma14102569

**Published:** 2021-05-15

**Authors:** Monika Dobrzyńska-Mizera, Monika Knitter, Salvatore Mallardo, Maria Cristina Del Barone, Gabriella Santagata, Maria Laura Di Lorenzo

**Affiliations:** 1 Institute of Materials Technology, Polymer Division, Poznan University of Technology, Piotrowo 3, 61-138 Poznan, Poland; monika.knitter@put.poznan.pl; 2 National Research Council (CNR), Institute of Polymers, Composites and Biomaterials (IPCB), c/o Comprensorio Olivetti, via Campi Flegrei, 34, 80078 Pozzuoli, NA, Italy; salvatore.mallardo@ipcb.cnr.it (S.M.); cristina.delbarone@ipcb.cnr.it (M.C.D.B.); gabriella.santagata@ipcb.cnr.it (G.S.); marialaura.dilorenzo@ipcb.cnr.it (M.L.D.L.)

**Keywords:** biocomposites, thermomechanical properties, thermal analyses, extrusion

## Abstract

Bio-based composites made of poly(L-lactic acid) (PLLA) and β-cyclodextrin/d-limonene inclusion complex (CD-Lim) were prepared by melt extrusion. Encapsulation of volatile d-limonene molecules within β-cyclodextrin cages was proven to be a successful strategy to prevent evaporation during high-temperature processing. However, small amounts of limonene were released upon processing, resulting in the plasticization of the polymeric matrix. Morphological analysis revealed good dispersion of the filler, which acted as a nucleating agent, favoring the growth of PLLA crystals. The composites′ lowered glass transition temperature upon the addition of CD-Lim was also proved by thermomechanical analysis (DMA). Moreover, DMA revealed constant stiffness of modified materials at room temperature, which is crucial in PLLA-based formulations.

## 1. Introduction

Poly(L-lactic acid) (PLLA) is a thermoplastic aliphatic polyester produced from annually renewable resources, which is biodegradable, compostable, and biocompatible [1]. Among the various biodegradable and bio-based polymers, PLLA has the highest potential to replace traditional petroleum-based polymeric materials. PLLA is currently used in multiple industrial fields, including biomedicine, packaging, automotive, and electronic industries [2], with considerable growth expected in applications where a relatively short lifetime can be forecasted, such as food packaging [3].

Significant research efforts have been devoted to promoting PLLA and other biodegradable polymers for active food packaging [4]. This type of packaging incorporates components that can release or absorb substances into or from the packaged goods or that can grant a tailored headspace composition within the package [5]. Introducing active ingredients towards specific pathogens in the package′s formulation also reduces the need for synthetic food additives and has a positive health impact [6]. Among the various additives currently used as food preservatives and active ingredients in food packaging, essential oils (EOs) play a prominent role as antifungal and antiviral agents [7,8,9,10,11]. EOs are natural volatile substances produced by plants and are hence excellent candidates to be added to a packaging that aims to not only be active but also bio-based, like a PLLA-based packaging [12].

However, incorporation of essential oils into PLLA is not a straightforward procedure. The boiling point of most essential oils is well below the melt processing temperature of PLLA, which prevents direct melt mixing of the components and hinders further processing of the material aimed at obtaining films by casting, compression molding, or extrusion. Moreover, most EOs are miscible with PLLA and plasticize the polymer, with marked and sometimes unwanted side effects on material properties, such as migration from the polymer matrix [13,14,15,16]. A possible route to overcome these problems is to incorporate essential oils within cyclodextrins, cage-like cyclic oligosaccharides produced from starch, a renewable natural material [17]. Cyclodextrin (CD) molecules can form inclusion complexes with various non-polar, aliphatic, and aromatic compounds. They are able to incorporate antimicrobials agents and, as a result, control their release, influence the product’s quality, and prolong its suitability for consumption [18]. The capability to embody fine particles within their hollows led to the considerable usage of cyclodextrins in a vast application area, such as pharmaceutical, food, and cosmetic industries. The inclusion of a volatile guest particle strongly decreases its diffusivity and volatility, hence protecting it from oxidation and thermal decomposition [19,20]. Thus, the bioactive compounds included inside the hydrophobic CDs cage can be feasibly carried and released by polymer matrices.

In a previous manuscript, we reported that d-limonene (4-isopropenyl-1-methylcyclohexane), the antimicrobial component of citrus peels, can be successfully encapsulated within β-CD [21], the CD modification made of seven glucopyranose units [17]. The incorporation inside β-CD enhanced d-limonene’s (Lim) thermal stability allows its melt processing with poly(butylene succinate) (PBS) [21].

d-limonene is a natural antimicrobial agent [22,23,24] itemized in the Code of Federal Regulations and commonly identified as a harmless flavoring agent and food preservative [25]. Its antibacterial activity has been proven with various types of food-related microorganisms, such as *Staphylococcus aureus*, *Listeria monocytogenes*, *Salmonella enterica*, *Saccharomyces bayanus*, etc. [26,27].

Based on the successful inclusion of the CD-Lim complex within PBS, which allowed development of fully biobased and biodegradable composites, we prepared novel composites based on PLLA as polymer matrix and CD-Lim as a dispersed phase. Inclusion of CD-Lim within PLLA by melt-processing, instead of PBS, is challenging since PLLA has a much higher melting temperature than PBS (160–200 °C vs. 115 °C), which may result in sizable evaporation of volatile Lim (boiling point 176 °C) upon melt processing. Therefore, considerable care was taken to prepare the composites, including proper choice of the PLLA grade and tailored processing conditions. The selected PLLA grade, detailed below, has 4% of d-isomer units, which implies a lower melting temperature than more stereoregular grades [1], hence, it can be melt-processed at lower temperatures. Details on the preparation and chemico-physical properties of PLLA/CD-Lim composites are reported in this manuscript, where it is shown that the proposed strategy allows incorporation of d-limonene within PLLA. The results presented here serve as basis to test suitability of the prepared materials for food packaging, with data detailed in a forthcoming manuscript [28] where mechanical, barrier, optical, and antibacterial/antifungal properties of PLLA/CD-Lim films are presented and discussed.

## 2. Materials and Methods

A poly(L-lactic acid), LX175, abbreviated PLLA, with MFR 6 g/10 min (210 °C, 2.16 kg), and containing 4% of d-isomer units in the chain, was kindly provided by Corbion (The Netherlands). β-cyclodextrin (CD) with a purity ≥99%, provided by Cyclodextrin Shop, was used after drying. A technical d-limonene grade (≈90% purity), abbreviated Lim, was supplied by Sigma Aldrich.

An inclusion complex of β-cyclodextrin and d-limonene was obtained via precipitation method [21]. First, 420 g of cyclodextrin was dissolved in 4200 mL of solution of ethanol and water (1:2) at 55 °C. d-limonene dissolved in ethanol (10% *v/v*) was gently poured to the heated CD solution under constant stirring, which was then held for an additional 4 h at room temperature. Then, the mixture was kept at 4 °C for 24 h. The filtrated CD-Lim complex was dried at 50 °C (24 h) and further at 25 °C (24 h). As reported in [21], this procedure leads to 7 wt% of d-limonene in β-cyclodextrin.

All materials were dried under vacuum at 50 °C for 24 h before processing. PLLA pellets were mixed with CD-Lim complex in a Retsch GM 200 rotary mixer (Haan, Germany) for 3 min at 2000 rpm. Based on results detailed in [21], composites containing a sizable amount of d-limonene, sufficient to ensure antimicrobial properties [28] were prepared. Mixing of the materials with various CD-Lim amounts (0–30 wt%) was carried out with a Zamak corotating twin-screw extruder (Skawina, Poland) operated at 190 °C and 60 rpm. The extruded rod was pelletized upon cooling in air. Compositions were selected to have a sizable amount of Lim in the composites, with PLLA/CD-Lim composites containing 20 and 30 wt% of CD-Lim, as summarized in Table 1.

The composites were compression-molded with a Collin Laboratory Forming Press P 200 E (Chengdu, China) at a temperature of 190 °C for 3 min, without any pressure applied to allow complete melting. After this period, a load of about 200 bar was applied for 3 min, then the samples with a thickness of 1 mm were air-cooled to room temperature. A similar procedure was followed to produce thin films with a thickness of approx. 150 μm. In this case, compression molding was conducted at 175 °C without any pressure, followed by an application of a load of about 10 bar for 1 min, and further 200 bar for another 1 min. Finally, the samples were air-cooled to room temperature.

Thermal properties were investigated with a Netzsch DSC 204 F1 Phoenix^®^ (Selb, Germany) apparatus, using aluminum crucibles and approximately 3 ± 0.5 mg samples under nitrogen flow. High purity standards were used to calibrate the instrument, including indium, tin, bismuth, zinc, and aluminum. The indium melting enthalpy was used for energy calibration. All the samples were heated to 200 °C and held in a molten state for 5 min, followed by cooling to −60 °C at heating and cooling rates of 10 °C min^−1^.

TGA analyses were performed in the temperature range between 30 and 600 °C, at a heating rate of 10 °C min^−1^, under a nitrogen atmosphere using a Netzsch TG 209 F1 apparatus (Selb, Germany) calibrated by analyzing several standards, including In, Sn, Bi, Zn, Al, and Ag. The decomposition onset temperature, *T*_o_, of approximately 10 mg samples, was determined at the intersection of tangents to two branches of the thermogravimetric curve [29]. Each measurement was preceded by an empty pan run, and subtracted from each thermogram to correct instrumental drift.

Dynamic mechanical analysis was carried out using an Anton Paar model Physica MCR 301 rheometer (Graz, Austria) in torsion mode. With dimensions of 10 × 4 × 50 mm, the composites underwent analysis in a temperature range between 25 and 130 °C at a frequency of 1 Hz and a heating rate of 2 °C min^−1^.

Cryogenically fractured cross-sections of PLLA-based composites were analyzed using a Quanta 200 FEG, 338 FEI scanning electron microscope (Thermo Fisher Scientific, Eindhoven, The Netherlands). SEM microphotographs were collected at room temperature and a voltage of 20 kV. Before analysis, surfaces of the samples were sputter-coated with an 18 ± 0.2 nm layer of Au-Pd alloy by a MED 020 splattering device, Bal-Tec AG (Pfaffikon, Switzerland).

## 3. Results and Discussion

**Figure 1:**
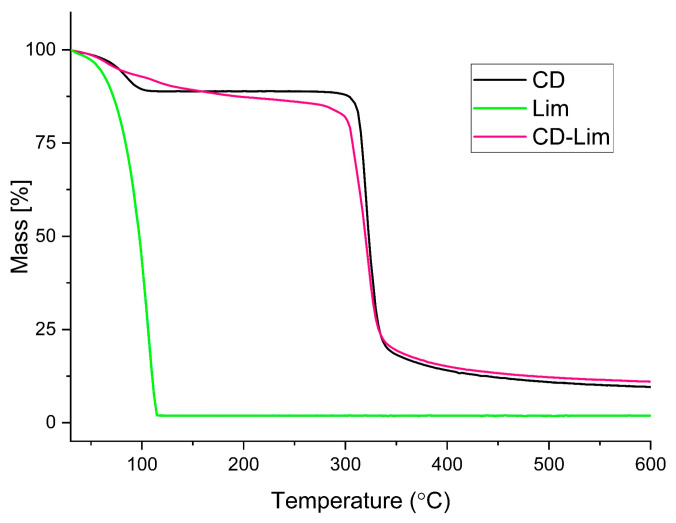
Thermogravimetric plots of β-cyclodextrin (black curve), d-limonene (green curve), and β-cyclodextrin/d-limonene inclusion complex (magenta curve), upon heating at 10 °C min^−1^ in nitrogen.

Our previous investigation demonstrated that d-limonene can be successfully included within β-cyclodextrin via precipitation, with proof of inclusion provided by infrared spectroscopy, ^13^C Nuclear Magnetic Resonance X-ray diffraction, and thermogravimetry [21]. Being the same procedure, only TGA analysis is repeated here as a tool to verify the efficacy of new sample preparation, as TGA can also provide quantitative analysis of the effective amount of Lim encapsulated within β-CD. TGA plots of pure β-cyclodextrin, d-limonene, as well as β-cyclodextrin/d-limonene inclusion complex are presented in Figure 1.

TGA curve of neat β-CD displays three significant mass loss steps. The first one, of 12% of the initial mass, was caused by the release of free and freeze-bound water up to about 110 °C. The second one starts around 300 °C and was related to a rapid mass loss of about 70%, due to depolymerization of cyclodextrin macromolecules and glucosidic rings, with the creation of carbonyl group and carbon-carbon double bonds [30]. The final thermal degradation stage occurred above 340 °C and revealed a slow thermal degradation of the residual char. In case of the CD-Lim, two degradation steps occurred in the range of 25–300 °C. The first one, ranging between 50–105 °C, reached a mass loss of about 8% due to the release of water and possibly also of a minor amount of Lim. Around 105 °C, a second, smaller mass decrease starts and finishes slightly below 300 °C, where the initial mass was cut to an overall 15% (7% in the second stage). This second step was caused by loss of the terpene, whose thermal stability is largely enhanced by encapsulation within β-CD cavity [30,31,32]. Moreover, TGA data of Figure 1 permits quantification of the amount of essential oil encapsulated within β-CD, which is about 7% *w/w*, in agreement with Ref. [21].

**Figure 2:**
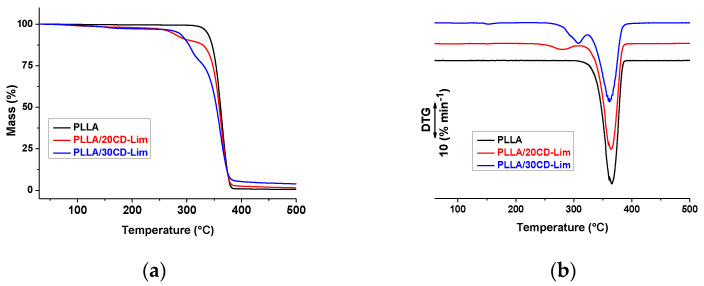
Thermogravimetric analysis of PLLA and PLLA/CD-Lim composites upon heating at 10 °C min^−1^ in nitrogen atmosphere: mass loss as a function of temperature (**a**) and the first derivative of the mass loss vs. temperature curves (**b**).

The TGA curves of PLLA and PLLA/CD-Lim sheets measured upon heating at 10 °C min^−1^ and normalized to the initial sample mass are presented in Figure 2a, with the first derivative curves shown as in Figure 2b. Plain PLLA exhibits a one-stage degradation process, where the polymer decomposes completely at about 380 °C, due to chain-end scission resulting in a constant lowering of molar mass [33,34,35,36]. Addition of CD-Lim complex to PLLA largely varies the mass-temperature profile, leading to a two-step degradation process due to different temperature ranges of degradation of the matrix and the filler. The lower thermal stability of CD-Lim, compared to plain PLLA, seen by comparison of the plots shown in Figure 1 and Figure 2, results in an enhanced onset of mass loss in the composites. The derivative plot of the composite containing 30% of the filler displays a small peak centered around 150–160 C, barely visible also for the sample containing 20% of CD-Lim, which possibly reveals evaporation of water, in agreement with Figure 1 (the samples were not dried before TGA analysis). This overlaps with Lim′s gradual release from CD upon heating of PLLA, completed upon depolymerization of β-CD, with the exact temperature being affected by composition. Possible interactions between the polar groups of β-CD surface and carbonyl groups of PLLA induce a joint impact on thermal decomposition kinetics, resulting in varied thermal stability of the composites depending on filler content. The delayed thermal degradation of CD-Lim in the sample containing a higher filler amount, revealed by the peak around 300 °C in the first derivative plots shown in Figure 2b, reveals a protective effect of the matrix against thermal degradation of the filler. Conversely, CD-Lim seems not to affect the thermal stability of PLLA, which occurs at temperatures where the filler is mostly decomposed.

**Figure 3:**
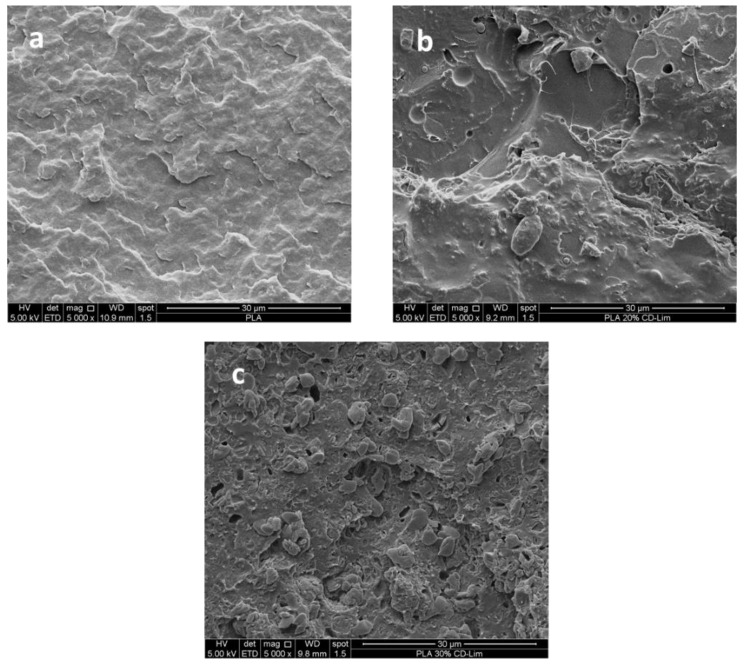
SEM micrographs of PLLA (**a**), PLLA/20CD-Lim (**b**), and PLLA/30CD-Lim (**c**) cryogenically fractured surfaces.

Morphology of PLLA/CD-Lim composites was investigated by scanning electron microscopy. The electron micrographs of fractured cross-sections of pure PLLA, PLLA/20CD-Lim, and PLLA/30CD-Lim composites are compared in Figure 3 at the same magnification. Figure 3a shows the fractured surfaces of compression molded, neat PLLA, which is smooth, as expected. The addition of 20 wt% of CD-Lim (Figure 3b) results in varied morphology, with the appearance of small voids and embedded particles homogeneously distributed along the whole surface of the sample. With the increase of filler content (30 wt%, Figure 3c), the electron micrograph shows several discrete microdomains distributed inside the polymeric matrix; some of them are agglomerated in small clusters. Agglomeration may be favored by the cyclodextrin hydrophilic surfaces and higher concentration inside the polymer matrix. It is worth noting that only a part of the filler particles were pulled out during the cryogenic fracture process; some of the particles remained attached to the matrix, suggesting compatibility between the components.

**Figure 4:**
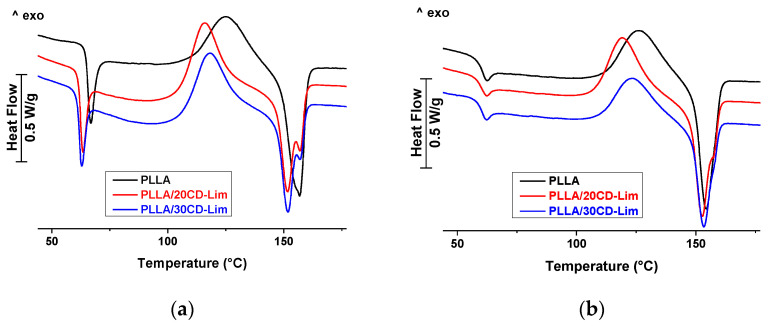
Heat flow rate plots of PLLA and PLLA/CD-Lim composites upon heating at 10 °C min^−1^: (**a**) heating scan of the compression-molded sheets and (**b**) heating scan of the same samples after cooling at 10 °C min^−1^. Exotherm upward.

Thermal analysis of PLLA and PLLA/CD-Lim composites are presented in Figure 4 as heat flow rate plots in a temperature function. Figure 4a reports the thermograms detailing the first heating scan of the compression molded sheets, whereas Figure 4b displays the second heating scan of the same samples after cooling at a constant rate of 10 °C min^−1^. The DSC thermogram of compression molded, plain PLLA displays a glass transition (*T*_g_) at 66 °C coupled with enthalpy relaxation, followed by a cold crystallization exotherm and a double melting peak. The latter is often observed in thermal analysis of PLLA and is linked to melting of two crystal modifications, named α′ and α [1,37,38]. Glass transition of the composites, centered at 62 °C for both analyzed compositions, slightly shifted to lower temperatures, due to partial release of d-limonene from β-CD cages to the PLLA matrix upon melt processing. The amount of Lim dissolved in PLLA was very similar in the two compositions (1.2–1.6 wt%, as quantified below) and results in a minor difference in plasticization, and practically overlapping *T*_g_s of the composites. It needs to be underlined that the measured *T*_g_ of the composites, which is 4 °C lower than in PLLA, well compares to literature data of PLLA/Lim formulations, as addition of much larger amounts (20 wt%) of Lim was reported to lead to a decrease of *T*_g_ of about 30 °C with respect to PLLA [39].

Above *T*_g_, the composites display a broad endotherm extending from the end of *T*_g_ to the onset of cold crystallization. This is caused by evaporation of a small fraction of Lim, which was released from the β-CD cages upon melt processing, and remained dissolved within the PLLA matrix, slightly plasticizing it. The enhanced molecular mobility above *T*_g_ allows release of the volatile component, resulting in a broad endotherm in the DSC plot.

The cold crystallization exotherm evidences an anticipated onset in the composites, compared to plain PLLA. This is linked to the varied crystallization window caused by plasticization, which shifts the phase transition to lower temperatures [40]. The initial slope of the cold crystallization peak increases with CD-Lim content, i.e., crystallization proceeds faster in the initial stage of the phase transition, as typically occurs in the presence of nucleating agents [41]. The multiple melting of PLLA, due to melting of α′- and α-crystals was only slightly varied in the composites, being determined by the cold crystallization temperature and crystal reorganization occurring upon heating [38].

Analysis of the weak endotherms between *T*_g_ and the onset of cold crystallization allows quantification of the amount of Lim that evaporates upon heating. Integration of these endothermic peaks leads to an enthalpy of 3.6 and 4.3 J g^−1^ for the samples containing 20 and 30 wt% of CD-Lim, respectively. Considering that enthalpy of vaporization of d-limonene is equal to 284 J g^−1^ [42], about 1.2 and 1.6 wt% of Lim evaporated upon DSC heating of the composites with 20 and 30 wt% of CD-Lim, respectively. This is the amount of d-limonene released from the β-CD cages upon melt processing, which remained dissolved within PLLA amorphous chain portions, plasticizing the polymer matrix. 

Cooling at 10 °C min^−1^ did not allow crystallization of the polymer, as expected for a PLLA grade containing 4% d-isomer units [37,38,43], and results in the subsequent heating, in a marked cold crystallization exotherm, followed by a melting endotherm of similar size, as seen in Figure 3b. Unlike the first heating, in the second heating scan, *T*_g_ seems to slightly increase with filler content, but with a minor variation that falls within experimental uncertainty. No endotherm of evaporation of Lim can be evidenced in the temperature range between *T*_g_ and the onset of cold crystallization, proving that all limonene that was initially dissolved within PLLA had already evaporated upon previous heating. This rationalizes the invariance of *T*_g_ with the composition seen in Figure 4b. It needs to be underlined that, similarly to the data shown in Figure 4a, cold crystallization of PLLA starts at lower temperatures in the composites compared to the plain polymer, confirming that CD-Lim can promote the onset of crystallization of PLLA.

**Figure 5:**
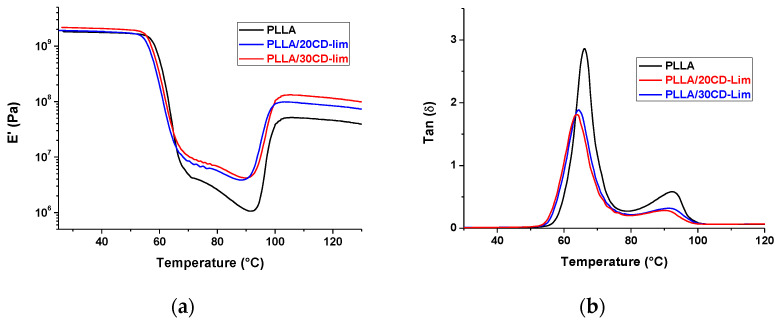
Storage modulus (E′) (**a**) and damping factor (tan δ) (**b**) vs. temperature of PLLA and PLLA/CD-Lim composites at a heating rate of 2 °C min^−1^ and frequency of 1 Hz.

Thermo-mechanical properties of PLLA/CD-Lim composites were also investigated. Figure 5 shows the storage modulus (*E*′) and damping factor (tan δ) of PLLA/CD-Lim composites. At low temperatures, all samples display a storage modulus of about 2 × 10^9^ Pa, as typical of glassy polymer, with slight variations among the grades close to the experimental uncertainty. By raising the temperature, glass transition occurs around 60 °C, as revealed by the sudden drop of *E*′ and the sharp peak detected in tan δ plots. In the composites, *T*_g_ takes place at slightly lower temperatures than plain PLLA [44,45], in agreement with DSC data of Figure 4a. Upon further increase of the temperature, at about 90 °C, PLLA chains gain sufficient mobility to cold crystallize, which results in a sizable rise in *E*′. Consequently, the segmental motion and molecular relaxation processes drastically reduced, as evidenced by the decreasing of tan δ both intensity and amplitude [46].

Cold crystallization has an anticipated onset in the composites compared to the plain polymer, which corroborates the knowledge gained by DSC analysis. The onset of crystallization was measured at about 90 °C by DMA upon heating at 2 °C min^−1^ (Figure 5a) and at about 100 °C by DSC upon heating at 10 °C min^−1^ (Figure 4a). The lower heating rate in DMA allows more time for chain ordering, favoring crystal nucleation and growth at lower temperatures, which results in an anticipated onset of cold crystallization [47]. After completion of crystallization, the storage modulus appeared to be sizably affected by the filler′s presence, as it increases with CD-Lim content. This effect is not seen at low temperatures below *T*_g_, where only minor differences in *E*′ values could be measured. At low temperatures, the plasticizing effect of limonene on PLLA matrix counterbalances the raised stiffness due to the presence of the filler. Upon heating, the small amount of d-limonene dissolved in PLLA evaporates, and only the reinforcing effect of the filler remains, causing sizable variation in the storage modulus with composition.

## 4. Conclusions

Composites prepared with PLLA and β-cyclodextrin containing d-limonene, all bio-based, were produced via twin-screw extrusion in different compositions. Encapsulation of d-limonene within β-cyclodextrin cavities greatly enhances the thermal stability of the essential oil to a level that allows its melt processing with PLLA, without marked loss of the essential oil. The results detailed above demonstrate that this is a successful strategy to incorporate d-limonene within PLLA via melt processing.

The composite films are slightly plasticized by small amounts (about 1.5 wt%) of d-limonene released from the β-CD cages upon melt processing. CD-Lim filler affects crystallization kinetics of PLLA, acting as an efficient nucleating agent, able to promote PLLA crystals growth. All formulations have comparable stiffness at room temperature, which varies with filler content at temperatures above *T*_g_. Morphological analysis of the cryogenically fractured samples display a homogeneous distribution of the filler within PLLA, with limited agglomeration observed only at high filler content. Moreover, the filler particles seem to have a good level of adhesion to the PLLA matrix.

The changes in thermal and thermo-mechanical properties and structure, detailed herein, largely influence tensile behavior as well as barrier and optical properties of the PLLA/CD-Lim composites, with results detailed in a submitted manuscript [28]. More importantly, it was proved that the obtained composites have antimicrobial properties, since when PLLA/CD-Lim films are put in contact with a wide range of bacteria and fungi, they can inhibit growth of these microorganisms. Therefore, the PLLA/CD-Lim composites appear to be promising materials for design of active food packaging films [28].

## Figures and Tables

**Table 1:** Symbols and mass concentrations of samples.

Designation	Mass Concentration (wt%)		
	PLLA	CD-Lim	Lim
PLLA	100	0	0
PLLA/20CD-lim	80	20	1.2
PLLA/30CD-lim	70	30	1.6
